# Secondary Metabolites, Antioxidant, and Antiproliferative Activities of *Dioscorea bulbifera* Leaf Collected from Endau Rompin, Johor, Malaysia

**DOI:** 10.1155/2021/8826986

**Published:** 2021-01-11

**Authors:** Muhammad Murtala Mainasara, Mohd Fadzelly Abu Bakar, Abdah Md Akim, Alona C Linatoc, Fazleen Izzany Abu Bakar, Yazan K. H. Ranneh

**Affiliations:** ^1^Faculty of Applied Sciences & Technology, Universiti Tun Hussein Onn Malaysia (UTHM), Hab Pendidikan Tinggi Pagoh, KM1, Jalan Panchor, Muar 84600, Johor, Malaysia; ^2^Faculty of Science, Department of Biological Sciences, Usmanu Danfodiyo University Sokoto, PMB 1046, Sokoto, Nigeria; ^3^Department of Biomedical Sciences, Faculty of Medicine and Health Sciences, Universiti Putra Malaysia, Seri Kembangan 43400, Selangor, Malaysia

## Abstract

Breast cancer is among the most commonly diagnosed cancer and the leading cause of cancer-related death among women globally. Malaysia is a country that is rich in medicinal plant species. Hence, this research aims to explore the secondary metabolites, antioxidant, and antiproliferative activities of *Dioscorea bulbifera* leaf collected from Endau Rompin, Johor, Malaysia. Antioxidant activity was assessed using 2,2-diphenyl-1-picrylhydrazyl (DPPH), ferric reducing antioxidant power (FRAP), and 2,2′-azino-bis-3-ethylbenzthiazoline-6-sulphonic acid (ABTS) assays, while the cytotoxicity of *D. bulbifera* on MDA-MB-231 and MCF-7 breast cancer cell lines was tested using 3-(4,5-dimethylthiazol-2-yl)-2,5-diphenyl tetrazolium bromide (MTT) assay. Cell cycle analysis and apoptosis were assessed using flow cytometry analysis. Phytochemical profiling was conducted using gas chromatography-mass spectrometry (GC-MS). Results showed that methanol extract had the highest antioxidant activity in DPPH, FRAP, and ABTS assays, followed by ethyl acetate and hexane extracts. *D. bulbifera* tested against MDA-MB-231 and MCF-7 cell lines showed a pronounced cytotoxic effect with IC_50_ values of 8.96 *μ*g/mL, 6.88 *μ*g/mL, and 3.27 *μ*g/mL in MCF-7 and 14.29 *μ*g/mL, 11.86 *μ*g/mL, and 7.23 *μ*g/mL in MDA-MB-231, respectively. Cell cycle analysis also indicated that *D. bulbifera* prompted apoptosis at various stages, and a significant decrease in viable cells was detected within 24 h and substantially improved after 48 h and 72 h of treatment. Phytochemical profiling of methanol extract revealed the presence of 39 metabolites such as acetic acid, n-hexadecanoic acid, acetin, hexadecanoate, 7-tetradecenal, phytol, octadecanoic acid, cholesterol, palmitic acid, and linolenate. Hence, these findings concluded that *D. bulbifera* extract has promising anticancer and natural antioxidant agents. However, further study is needed to isolate the bioactive compounds and validate the effectiveness of this extract in the In *in vivo* model.

## 1. Introduction

Millions of mortalities were reported globally as a result of cancer, and the numbers of new cases are expected to increase in the future [1]. Breast tumour is the second highest cancer recorded worldwide and the most common cause of cancer death among women. It has been ascertained that about 25% of all new identified cancer case is breast cancer, which accounts for 15% of deaths in women each year [2, 3].

Breast cancer is a commonly diagnosed cancer in Malaysia, having a standardized age rate of 47.4 per 100,000 [4, 5]. The National Cancer Registry of Malaysia (NCR) records that 21,773 Malaysians who are perceived with cancer and approximations show that unregistered cases have reached about 10,000 cases every year. It is likely that by 75 years old, ¼ of Malaysians will be diagnosed with cancer [6]. Therefore, discovering new drugs with minimal toxicity is a broad scientific challenge, and there is an immediate need to search for a new agent to mitigate the menace by using alternative evidence-based herbal medicines.

**Figure 1:**
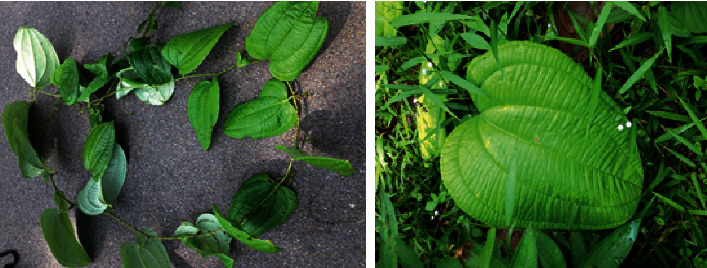
*D. bulbifera* plant (leaves).

*Dioscorea bulbifera* (Figure 1) is a member of the family Dioscoreaceae, categorised in the order Dioscoreales and genus *Dioscorea*. It is usually referred to as air potato, air yam, or bulbil-bearing yam. It is a prolific climbing plant indigenous to Southern Asia and West Africa, mostly found along forest edges. The direction of circular twinning had been reported in *D. bulbifera* and can grow to a height of 12 m. It has shiny green leaves that are alternate with a long leafstalk. Resembling true yam leaves, *D. bulbifera* has an annual vegetative cycle. Hence, this study was directed toward discovering secondary metabolites, antioxidant, and antiproliferative effects of *D. bulbifera* leaf extract from Kampung Peta, Endau Rompin, Mersing, Johor, Malaysia.

## 2. Materials and Methods

### 2.1. Sample Collection and Preparation

*D. bulbifera* fresh leaves were collected at Kampung Peta, Johor, Malaysia, on 5^th^ May 2017 with the permission of Perbadanan Taman Negara Johor (PTJN) and with full observation of rules and regulations on collection practices of medicinal plants as laid down by the World Health Organisation (WHO) collection of plant materials [7]. Identification was made, and the voucher specimen was deposited in the Herbarium of Centre of Research for Sustainable Uses of Natural Resources (CoR-SUNR), Faculty of Applied Sciences and Technology, Universiti Tun Hussein Onn Malaysia. Fresh sample was cleaned using tap water to eliminate impurities or soil debris. The sample was crushed to a fine powder with an electric blender after shade drying at room temperature and kept in a zip lock bag in a freezer (−20°C) for further analysis [8, 9].

### 2.2. Sample Extraction

Methanol, ethyl acetate, and hexane solvents were used for the extraction of plant samples by successive maceration methods as described by Bhunu et al. [10] with some modifications. The mixture was filtered using a vacuum filter and then evaporated using a rotary evaporator where the yield of extracts was found; hexane (6.22%), ethyl acetate (8.63%), and methanol (20.6%) were used. The extract was later used to identify its phytochemicals, antioxidant, and antiproliferative effects.

### 2.3. Determination of Antioxidant Activities

#### 2.3.1. 2,2-Diphenyl-1-picrylhydrazyl (DPPH) Assay

DPPH scavenging activity of the sample was determined according to Bakar et al. [11] and Hassan and Bakar [12]. Briefly, a methanol solution of DPPH (0.3 mM) was added into the samples (2.5 mL). The extract was kept in a light protected place at room temperature for 30 min after mixing vigorously. The absorbance was read at 518 nm. The following equation was used for calculating the scavenging or antioxidant activity (AA):

(1) $$\begin{array}{c} \text{AA}\left(\%\right)=\frac{\text{Absorbance }\left(\text{sample}\right)-\text{absorbance }\left(\text{empty sample}\right)}{\text{absorbance }\left(\text{control}\right)}\times 100\text{.} \end{array}$$

#### 2.3.2. Ferric Reducing Antioxidant Power (FRAP) Assay

FRAP assay was conducted according to Dusuki et al. [13] with some modifications. The FRAP mixture was formed by mixing 300 mM acetate buffer with 3.6 pH, 2.5 mL of 10 mM TPTZ solution in 40 mM HCl, and 0.25 mL of 20 mM FeCl3, and the substances were tested in 0.1 mL methanol, ethyl acetate, and hexane or methanol. Plain reading was recorded at 593 nm using a test tube with extra 3 mL of FRAP reagent. Plant extract (100 *µ*L) and distilled water (300 *µ*L) were supplemented to the test tube. Using a spectrometer, after 30 min of incubation, absorbance was read at 593 nm. Ferric reducing capacity in 1 g of dried sample (mM/g) was measured as mM for the final result. All tests were run in triplicate.

#### 2.3.3. 2,2′-Azino-bis-3-ethylbenzthiazoline-6-sulphonic Acid (ABTS) Assay

ABTS assay was carried in line with Re et al. [14] with some modifications. Reacting of ABTS solution (7 mM) with 2.45 mM of potassium persulfate produced the preformed radical monocation of ABTS. The solution was kept in the dark and left to stand for 15 h at room temperature. To obtain the units of absorbance at 734 nm, the solution was diluted with either methanol, ethyl acetate, or hexane; aliquot for every sample (200 *µ*L) was added to 2000 *µ*L of ABTS. Using a spectrometer, the absorbance was monitored for 5 min at 734 nm. Blank solvents were run appropriately in every assay. The proportion of inhibition was calculated against control and matched to a vitamin C standard curve (10–100 mm), and the percentage was used to express the radical scavenging activities.

### 2.4. Anticancer Activities

#### 2.4.1. Cell Culture Condition

MCF-7 and MDA-MB-231 breast cancer cell lines were purchased from American Type Culture Collection (ATCC). The cells were maintained in Roswell Park Memorial Institute (RPMI) 1640 media supplemented with L-glutamine, 1% penicillin-streptomycin, and 10% fetal bovine serum in a humidified atmosphere of 5% carbon dioxide (CO_2_) at 37°C.

#### 2.4.2. Extracts Preparation

To solubilise the plant extract, dimethyl sulphoxide (DMSO) at a concentration of 10 mM which was kept at 4°C under light protection was used. DMSO concentration that does not exceed 1% was used for the whole experiment. Fresh complete culture media were diluted with 20 mg/10 mL of standard drug (doxorubicin), and the extract was serially diluted to the cell plates at varying concentrations from 100 *µ*g/mL to 1.5625 *µ*g/mL.

#### 2.4.3. 3-(4,5-Dimethylthiazol-2-yl)-2,5-diphenyl Tetrazolium Bromide (MTT) Assay

Following a procedure described by Rahmat et al. [15], 6.9 × 10^5^ of MDA-MB-231 and MCF-7 cells were plated in 96-well plate and grown in 200 *µ*L complete growth media (CGM) and treated for 24 h, 48 h, and 72 h, respectively. IC_50_ of the extract was added in 96-well plates in serial dilution (100 *μ*g/mL–5.625 *µ*g/mL), and then, 20 *μ*L of MTT was added into each well and nurtured for 4 h. Afterward, to liquefy and solubilize the colored crystals, 100 *μ*L of DMSO was added into each well, and absorbance was read at 570 nm using an ELISA reader (AWARENESS-State Fax, USA). The following formula was used to determine the cytotoxicity of the extract:

(2) $$\begin{array}{c} \text{Cytotoxicity \% }=\;\frac{\text{optical density of sample}}{\text{optical density control}}\times 100\text{.} \end{array}$$

For the suppression concentration (IC_50_), the quality of sample that readily inhibits cell division by half was determined explicitly for every cell multiplying curve.

#### 2.4.4. Cell Cycle Analysis by Propidium Iodide (PI) Staining

Cell cycle analysis was carried out with some modifications as described by Queiroz et al. [16]. Cells (1 × 10^6^) were nurtured for 24 h and subsequently treated for 24 h, 48 h, and 72 h using crude extracts at the IC_50_ values. Altogether, the detached and adhering cells were reaped and reassigned in a disinfected centrifuge tube. The cells were then centrifuged (1,200 rpm) for 15 min at 4°C. Cold phosphate buffered saline (PBS) was used to wash the cells where the cells were resuspended with 0.5 mL of cold PBS, and ice-cold ethanol (70%) was then added to the cell suspension and incubated at −20°C for 2 h. Then, the ethanol was expelled from the sample after centrifugation. The cells that have been rinsed twice using cool PBS previously were stained with propidium iodide (500 *μ*L of 10 *μ*g/mL) and RNase (100 *μ*g/mL) at room temperature for about 30 min. Dispersion of the cell cycle was analysed using flow cytometry.

#### 2.4.5. Annexin V/PI Apoptosis Assay

This method was performed according to Tor et al. [17]. Briefly, cells were plated in 6-well plates and treated with IC_50_ values for 24 h, 48 h, and 72 h. The viability of the cells was measured using an annexin V-FITC/PI kit, following the manufacturer's procedure. Both treated and control cells were centrifuged and washed twice using PBS. At room temperature, cell staining was carried out using FITC annexin V (5 *µ*L) and 10 *µ*L PI for 15 min, and cell death was also analysed using flow cytometry.

### 2.5. Gas Chromatography-Mass Spectrometry (GC-MS) Analysis

The selected crude extract was analysed using gas chromatography (GC-MS-2010 Plus-Shimadzu) in order to determine the secondary metabolites present. The segment (30.0 m length, 0.25 mm ID, and 0.25 *μ*m thickness) was set at 50°C for 4 min which was then expanded to 300°C at the rate of 3°C/min, and after that supported for 10 min. The temperature at the injector was set at 250°C, and the volume was set at 0.1 L. The present helium rate of the bearer gas was set at 1 mL/min with an aggregate run span of an hour. Electron ionization and mass spectra were performed at 70 eV and between the range of 40–700 m/z [18].

### 2.6. Statistical Analysis

All the data were presented as mean ± standard error of mean (S.E.M). The data were statistically analysed by one-way ANOYA, followed by Dunnett's posthoc using Prism version 15.0. The level of statistical significance was set at *p* < 0.05.

## 3. Results

### 3.1. Antioxidant Activity of the Plant Extracts

Since one antioxidant assay is not sufficient to provide an adequate picture of the scavenging activities, *D. bulbifera* leaf extracts were subjected to several antioxidant assays. Antioxidant potential of *D. bulbifera* leaf extracts was evaluated using DPPH, FRAP, and ABTS assays (Table 1). For the DPPH assay, the methanol extract of *D. bulbifera* had the highest scavenging activity with the value of 79.0 ± 0.31 %, followed by the ethyl acetate extract with 23.2 ± 0.05 % and hexane extract with 11.5 ± 0.31 %, respectively. The same observation was also found in the FRAP assay where the methanol extract demonstrated highest reducing capacity with a value of 65.6 ± 0.35 mM Fe^2*+*^/g, followed by ethyl acetate with 59.5 ± 0.10 mM Fe^2 +^/g and hexane extract with 14.9 ± 0.05 mM Fe^2*+*^/g, respectively. Whereas for the ABTS assay, the methanol extract also had the highest scavenging activity with a value of 31.34 ± 2.06 mg AEAC/g, followed by the ethyl acetate extract with 10.98 ± 0.64 mg AEAC/g and hexane extract with a value of 9.50 ± 0.48 mg AEAC/g. Plant phenolics are a significant group of compounds that serve as major antioxidants or free radical scavengers. Hence, in this analysis, the observed high free radical scavenging behavior of the methanol extract may have accounted for its polarity for the radical scavenging ability of the plant extracts. The radical scavenging activities may be due to the presence of some flavonoids with a free hydroxyl group, capable of donating hydrogen and electron [12].

The findings showed that the plant extracts had substantial disparities in their ability to compare ascorbic acid, which may also be ascribed to various characteristics, reaction, and mechanisms. Organic media can be used in solubilising DPPH radical chromogens. A strong positive correlation (*R*^2^ = 0.9399, *p* < 0.05) was observed between the activities. There was also a similar relationship in FRAP, where a strong positive linear correlation (*R*^2^ = 0.8143, *p* < 0.05) was found. FRAP also correlates with ABTS• +  (*R*^2^ = 0.87), whereas ABTS• +  showed a stiff positive association to VCEAC (*R*^2^ = 0.6784). The practical difference in these comparisons was DPPH•, which also correlates poorly with ABTS (*R*^2^ = 0.53).

### 3.2. Anticancer Activities of *D. bulbifera*

#### 3.2.1. 3-(4,5-Dimethylthiazol-2-yl)-2,5-diphenyl Tetrazolium Bromide (MTT) Assay

Cytotoxic activity of the plant extracts against the breast cancer cells lines was assessed using MTT assay. The IC_50_ values of extracts on the viability of cancer cells after 24 h, 48 h, and 72 h of incubation were evaluated. IC_50_ values were determined which lower IC_50_ values signifying a higher antiproliferative activity. All the three extracts tested have demonstrated significant and effective antiproliferative activities in both dosage and time-dependent manner.

The IC_50_ values of the extract on the viability of cells in MCF-7 after 24 h, 48 h, and 72 h of incubation were 41.17 *μ*g/mL, 15.71 *μ*g/mL, and 11.53 *μ*g/mL, respectively, while the standard drug, doxorubicin, has shown the potent antiproliferative effect on the tested cell line with the IC_50_ values of 5.87 *μ*g/mL, 3.23 *μ*g/mL, and 1.98 *μ*g/mL for 24 h, 48 h, and 72 h, respectively. For the MDA-MB-231 cell line, the methanol extract of *D. bulbifera* had IC_50_ values of 4.29 *μ*g/mL, 1.86 *μ*g/mL, and 1.23 *μ*g/mL, respectively, at 24 h, 48 h, and 72 h. On the other hand, doxorubicin displayed potent cytotoxicity against the tested cell line with IC_50_ values of 11.21 *μ*g/mL, 8.1 *μ*g/mL, and 3.07 *μ*g/mL for 24 h, 48 h, and 72 h, respectively.

#### 3.2.2. Cell Cycle Arrest

**Figure 2:**
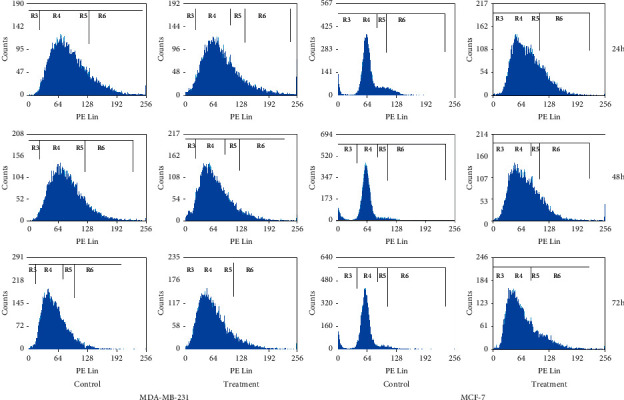
Flow cytometric scans of treated and untreated (control) cells.

Using the IC_50_ concentration from MTT assay for 24 h, 48 h, and 72 h, cell cycle analysis was evaluated after exposure of *D. bulbifera* methanol extract (Figure 2). In MCF-7, there was a momentous arrest at sub-G_1_, at 24 h–72 h, and the number of cells at S and G_2_/M phases and the number of cells at G_0_/G_1_ were reduced substantially. In MDA-MB-231, there was a significant arrest at sub-G_1_ and G_2_/M for 24 h, 48 h, and 72 h, and the number of cells in G_0_/G_1_ and the number of cells in G_2_/M were markedly reduced. At 48 h, there was also considerable arrest at sub-G_1_ and G_0_/G_1_, and the number of cells in S and the number of cells in G_2_/M phases were markedly reduced, and eventually at 72 h, there was a substantial arrest at sub-G_1_ and S, and the number of cells in G_0_/G_1_ and G_2_/M phases decreased significantly.

#### 3.2.3. Apoptosis

**Figure 3:**
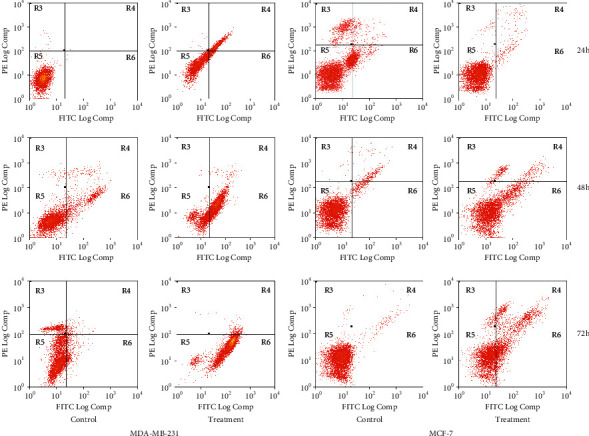
Annexin V FTIC of treated and untreated (control) cells.

Cell lines were treated, and the IC_50_ values for 24 h, 48 h and 72 h were obtained. The cells were stained with both FITC-conjugated annexin V and Pl. FACS was utilised to acquire the stained cell populace. Histograms from FACS investigation at each concentrate fixation are shown in Figure 2. Graphical presentation (Figure 3) shows the proportion of annexin-V-FITC and PI-stained cells at the different time of treatment, at an early stage of apoptosis, apoptotic, and late apoptotic cells. Plant extracts for the treatment were examined for 24 h, 48 h, and 72 h in cell lines.

Figure 3 shows the percentages of cell viability after treatment of *D. bulbifera* methanol extract in MCF-7. At the end of treatment (after 72 h), the percentage of feasible cells decreased ominously by 7.78%, while the percentage of cells increased by 3.99% at early apoptosis. The cell proportion also rises by 1.06% in the late apoptotic period. Nevertheless, the percentage of cells in early apoptosis decreased by 3.8% for the control culture at 72 h; the late apoptotic stage also decreased by 0.02%, while the number of viable cells increased by 8.02%. Although the proportion of cells in the group of live cells decreased significantly by 28.43% in MDA-MB-231 after 72 h of treatment, the percentage of cells in early apoptosis increased by 29.37%. Similarly, at the necrotic level, the proportion of cells increased by 41.46% but decreased by 0.74% in the late apoptotic period. However, for the control culture at 72 h, the proportion of cells with early apoptosis decreased by 4.14%, the late apoptotic stage also decreased by 14.62%, while the apoptotic period increased by 1.08%, and a number of viable cells also decreased by 11.92%.

### 3.3. Gas Chromatography-Mass Spectroscopy (GC-MS) Analysis

GC/MS investigation was conducted on *D. bulbifera* methanol extract. The peaks in the chromatogram were integrated and matched with the database spectra of recognised compounds stored in the GC-MS libraries of Pfleger–Maurer–Weber-drug, National Institute of Standard and Technology (NIST), WILEY229.LIB, Flavour, Fragrance, Natural and Synthetic Compounds (FFNSC1.3.lib), and Pesticides Library for toxicology (PMW_tox2).

**Figure 4:**
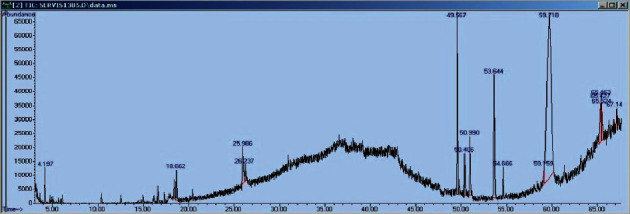
GC chromatograms of *D. bulbifera* methanol crude extract.

The result of *D. bulbifera* methanol extract revealed 50 peaks, with 45 compounds identified, representing 98.49% of the entire extract (Figure 4). The major among them were acetic acid (34.68%), n-hexadecanoic acid (14.89%), 1,2,3-propanetriol, 1-acetate (acetin) (7.28%), hexadecanoate <methyl-> (4.01%), 7-tetradecenal (Z)- (2.92%), glycerol alpha-monoacetate (2.80%), phytol (2.46%), octadecanoic acid (2.26%), cholesterol (2.10%), palmitic acid (1.35%), linolenate <methyl-> (1.32%), megastigmatrienone and 8-oxabicyclo-oct-5-en-2-ol, 1,4,4-trimethyl (1.28%) each, 1,2,3-propanetriol (1.23%), and 4,4,5,8-tetramethylchroman-2-ol (1.22%).

## 4. Discussion

Medicinal plants have been of great interest as a source of natural antioxidants used for health promotion such as anticancer properties. In this present study, the radical scavenging activities of *D. bulbifera* leaf extracts were quantified using DPPH, FRAP, and ABTS assays. All the extracts demonstrated scavenging of stable DPPH and ABTS as well as reducing activity in FRAP assay. The secondary metabolites, namely, phenolics and flavonoids, present in this species make it potential for antioxidant activities by functioning as reducing agents [15, 19–22]. Numerous antioxidants that are described to have therapeutic potential such as vanillic acid, isovanillic acid, epicatechin, and myricetin are essential bioactive compounds in *D. bulbifera*. Tubers of *D. bulbifera* have displayed higher scavenging activity, reducing power, and ferrous ion chelating due to its high content of polyphenols such as oxalic acid, citric acid, malic acid, and succinic acid. Thus, the secondary metabolites might be the key role players behind its biological activities [23]. The methanolic extract of *D. bulbifera* has been reported to demonstrate DPPH radical-scavenging activity [24]. Studies of antioxidants from different platinum–palladium bimetallic nanoparticles (PtNPs) made from *D. bulbifera* indicates that PtNPs could inhibit radical DPPH by up to 30.16%, while palladium (PdNPs) showed up to 28.97% of the activity. Despite this, Ptfor–PdNPs reveal 38.49% scavenging when compared separately with PtNPs and PdNPs. In addition, for Pt–PdNPs (56.71%), a synergistic enhancement activity against radical superoxide was also observed as contrasted and only PtNPs (31.87%) or PdNPs (27.1%).

Another study which was conducted by Li et al. [25] revealed that *D. bulbifera* and other *Dioscorea* species possessed high antioxidant activity as in the following order *D. bulbifera*, followed by *D. collettii*, *D. nipponica,* and *D. opposita*. This indicates that members of the genus *Dioscorea* have therapeutic potential due to high antioxidants contents. Comparable study was carried out by Ghosh et al. [26]; copper nanoparticles (CuNPs) synthesised by *D. bulbifera* showed high radical scavenging activities that are slightly lesser when compared with ascorbic acid. Vitamin C also showed 14.11% of superoxide scavenging activity while CuNPs showed 48.39% of the activity.

The results of the present study indicated that the methanolic extract of *D. bulbifera* was found to be the most cytotoxic extract in both cell lines due to the presence of plentiful active compounds, namely, diosgenin in yam, the edible tubers of *Dioscorea* spp. These results are compatible with the studies conducted by Li et al. [27] and Lee et al. [28]. Diosgenin has been shown to enhance metabolism of lipids, improve antioxidant activities, reduce glucose levels, and suppress inflammation [29]. Previous research presented that foods rich in diosgenin such as yam species (known as air potatoes) found to have a protective effect on myocardial I/R wound in rats due to apoptosis and necrosis and showed a cytotoxic effect on cancer cell lines [29–34].

It was reported by Nur and Nugroho [32] that *D. bulbifera* extracts demonstrated a cytotoxic effect on T47D cell lines. This research result indicated that the chloroform extract of the *D. bulbifera* leaves was stronger in inhibiting the growth of the cells than the methanol extract. Similarly, Ghosh and his colleagues [26] revealed that different nanoparticles Pton–PdNPs, PdNPs, and PtNPs made from *D. bulbifera* tuber extract exhibited the antiproliferative effect where Pt–PdNPs had been the most cytotoxic nanoparticles at concentration 10 *μ*g/mL. *In vivo* studies conducted on cytotoxic activities of the water extract, nonethyl acetate extract, ethyl acetate extract, ethanol extract, and diosbulbin B isolated from *D. bulbifera* showed that ethanol and ethyl acetate extracts reduced the lump weight in S180 and H22 tumour cells bearing mice while no such effect was observed in water and nonethyl acetate extracts [35].

In this study, cell cycle analysis was conducted in MCF-7 and MDA-MB-231 cell lines after treated with *D. bulbifera* methanol extract at IC_50_ concentration for 24 h, 48 h, and 72 h. At 24 h, the extract induced S and G_2_/M phases arrest in MCF-7 and G_0_/G_1_ and G_2_/M phases arrest in MDA-MB-231. At 48 h, growth in a number of the treated cells was observed in the sub-G_1_ phase in MCF-7 and sub-G_1_ and G_0_/G_1_ in MDA-MB-231, while significant decreases in the number of cells were observed in G_0_/G_1_, S, and G_2_/M in MCF-7 and S and G_2_/M in MDA-MB-231. At 72 h, *D. bulbifera* methanol extracts arrested MCF-7 at the sub-G_1_ phase and MDA-MB-231 at sub-G_1_ and S phases.

The result of the present study was similar with Srinivasan et al. [36] where diosgenin led to cell growth in the G_1_ phase, consisting of 72% of cells in G_1_ at 24 h and 82% and 76% at 48 h and 72 h posttreatment, respectively, compared to untreated cells. Similar observation was found in diosgenin-treated MDA-MB-231 cells where there was aggregation of cells in the G_1_ process occurred (82% at 24 h, 81% at 48 h, and 71% at 72 h). In addition, Moalic et al. [37] found that cells treated with diosgenin suppressed 1547 cell proliferation rate after 12 h along with a significant accumulation of cells in the G_1_ phase which was increased at 24 h. Consequently, the fraction of S phase cells decreased at 12 h. A sub-G_1_ population, typically associated with apoptotic cells, appeared at 48 h when compared to controls. According to a similar study conducted by Wang and Weller [38], HepG2 cells treated with different concentrations of protodioscin (bioactive component in *D. collettii*) for up to 24 h, accumulated mainly in the G_2_/M phase in a dose-dependent and time-dependent manner with consequent increase in the sub-G_1_ phase of cell cycle. Moreover, diosgenin isolated from *D. bulbifera* was reported to induce S-phase arrest at a concentration of 13 *µ*M in DU-145 prostate cancer cells. At 24 h of incubation phase, S arrest was relatively substantial, but at 48 h of incubation, there was no such effect, and arrest was not found to be important in MCF-7 compared to DU-145 [39].

In 2005, Liu et al. [40] reported that NB4 cells treated with diosgenin resulted in the enlargement of cell size, and arrest was made at G_2_/M. Diosgenin increased the p53 levels in NB4 cells, indicating its importance in controlling cell cycle arrest. Flow cytometric sub-G_1_ analysis showed that a dramatic hypodiploid number of K562 cells appeared after treatment with diosgenin for 48 h along with DNA fragmentation. Another research carried out by Hsu et al. [41] presented that treatment of squamous cell carcinoma-25 (SCC-25) with red mold *Dioscorea* cell cycle for 24 h caused arrest at the G2/M phase. This effect was also connected to the repression of CDK1 and cyclin B1 mRNA levels, ensuring cell proliferation inhibition.

On comparative research by Liu and his colleagues [40], it was found that dioscin fundamentally hinders multiplication of C6 glioma cells at the S stage because of an expansion in a few cells in the stage following expanding dosages of dioscin increment in sub-G_1_ arrest. Demonstrating that dioscin caused cell cycle capture at the S stage, G_0_/G_1_ stage decline in a few cells was observed. It has been recently detailed that dioscin restrained ROS creation, brought about DNA harm, and made cell cycle captured at the S stage, when contrasted and untreated, gathering the extent of the G_0_/G_1_ stage decreased from 61.38% to 35.43%, and S stage increased from 30.62% to 59.77% by 5.0 mg/ml of dioscin while the extents of G_2_/M stage had small changes. These findings showed that dioscin was able to block human HEp-2 and TU212 cells at the S stage [42].

A study performed by Li et al. [27] showed that diosgenin treatment in focus subordinate caused increment of G_2_/M stage cell population in Bel-7721, SMMC7721, and HepG2 HCC cells, suggesting that diosgenin could arrest the cell cycle in the G_2_/M stage because of the way that extent of G_2_/M stage cells with expanded in centralization of diosgenin. Lee and his colleagues [28] presented the antiproliferative impacts of diosgenin from yam (*D. pseudojaponica*) on malignant growth (MCF-7, A 549, and Hep G2) and typical (HS68 and clone 9) cells. The outcome demonstrated that diosgenin from *D. pseudojaponica* hindered MCF-7 cells multiplication through G_0_/G_1_ arrest.

The importance of the apoptosis idea for oncology lies in its being a directed marvel subject to incitement and hindrance. Even though little was thought about how settled helpful operators for disease influence its introduction, it appears to be sensible to propose that more prominent comprehension of the procedures included may prompt the advancement of enhanced treatment regimens [43]. Apoptosis is a physiological process of cell death that is in charge of the destruction of cells in normal tissues; it likewise occurs in different pathologic settings. Morphologically, it involves quick build up and sprouting of the cells with the arrangement of membrane-enclosed apoptotic bodies containing well-preserved organelles which are phagocytosed and digested by nearby resident cells [44]. Generally, apoptosis involved two central pathways; the first one is the stimulation of death receptors (DRs) in the tumour necrosis factor (TNF) superfamily, and the second one is the mitochondrial pathway initiated by Bcl-2 family proteins [45].

Several drugs that are used for cancer treatment have been appeared to cause apoptosis in quickly dividing normal cell numbers and tumours. Consequently, enhanced apoptosis is liable for a significant number of the antagonistic impacts of chemotherapy and tumour regression. Clear understanding of the procedures involved might lead to the improved treatment regimen. Hence, the realization of anticancer drugs that mediated the therapeutic impact by activating apoptosis is an additional necessary outcome [44].

Annexin-V-FITC/PI-flow cytometry analysis ascertained the induction of apoptosis by plant crude in both MCF-7 cells and MDA-MB-231. Distinctive biochemical features characterise apoptosis in the removal of damaged cells or tumour cells without causing irritation. The commencement of enzymatic and catabolic procedures in apoptosis in this manner empower cell morphological changes, for example, externalization of the plasma layer, phosphatidylserine (PS), shrinking of the cells, blebbing in cell membrane, condensation of chromatin, nuclear fragmentation, and apoptotic bodies formation [46–49].

## 5. Conclusion

In conclusion, the effort presented herein has proved that *D. bulbifera* methanol extract had resilient antiproliferative activity when compared with a standard drug. The antioxidant activities shown by methanol, ethyl acetate, and hexane extracts of the plant were significant compared with ascorbic acid, indicating the potential of the leaves of this species as natural antioxidants. Hence, antiproliferative activities demonstrated by leaf extracts authenticate the old style uses of this plant against various diseases of breast cancer inclusive.

## Figures and Tables

**Table 1:** Antioxidants activities of *D. bulbifera* leaves extract.

Sample name	DPPH (%)	FRAP (mM/g)	ABTS (mg AEAC/g)
Ascorbic acid	97.0 ± 0.21	76.5 ± 0.5	47.1 ± 0.81
Methanol extract	79.0 ± 0.31	65.6 ± 0.35	31.34 ± 2.06
Ethyl acetate extract	23.2 ± 0.05	59.5 ± 0.10	10.98 ± 0.64
Hexane extract	11.5 ± 0.31	14.9 ± 0.05	9.50 ± 0.48

## Data Availability

The datasets generated and/or analysed during the current study are available from the first author upon request.
